# Energy Deficit Required for Rapid Weight Loss in Elite Collegiate Wrestlers

**DOI:** 10.3390/nu10050536

**Published:** 2018-04-26

**Authors:** Emi Kondo, Hiroyuki Sagayama, Yosuke Yamada, Keisuke Shiose, Takuya Osawa, Keiko Motonaga, Shiori Ouchi, Akiko Kamei, Kohei Nakajima, Yasuki Higaki, Hiroaki Tanaka, Hideyuki Takahashi, Koji Okamura

**Affiliations:** 1 Japan Institute of Sports Sciences, 3-15-1 Nishigaoka, Kita-ku, Tokyo 115-0056, Japan; hiroyuki.sagayama@jpnsport.go.jp (H.S.); keiko.motonaga@jpnsport.go.jp (K.M.); ouchi.shiori@gmail.com (S.O.); akiko.kamei@jpnsport.go.jp (A.K.); kohei.nakajima@jpnsport.go.jp (K.N.); hideyuki.takahashi@jpnsport.go.jp (H.Tak.); 2 Japan Society for the Promotion of Science, Kojimahi Business Center Building, 5-3-1, Kojimachi, Chiyoda-ku, Tokyo 102-0083, Japan; 3 National Institutes of Biomedical Innovation, Health and Nutrition, 1-23-1 Toyama, Shinjuku-ku, Tokyo 162-8636, Japan; yamaday@nibiohn.go.jp; 4 Faculty of Sports and Health Science, Fukuoka University, 8-19-1 Nanakuma, Jonan-ku, Fukuoka 814-0180, Japan; shiose@fukuoka-u.ac.jp (K.S.); higaki@fukuoka-u.ac.jp (Y.H.); htanaka@fukuoka-u.ac.jp (H.Tan.); 5 Department of Sports Wellness Sciences, Japan Women’s College of Physical Education, 8-19-1, Kitakarasuyama, Setagaya-ku, Tokyo 157-8565, Japan; takuya.osawa.jp@gmail.com; 6 Graduate School of Sport and Exercise Sciences, Osaka University of Health and Sport Sciences, 1-1, Asashirodai, Kumatori-cho, Sennan-gun, Osaka 590-0496, Japan; okamura@ouhs.ac.jp

**Keywords:** body composition, energy balance, weight loss, doubly labeled water, multi-component model

## Abstract

To determine energy density for rapid weight loss (RWL) of weight-classified sports, eight male elite wrestlers were instructed to lose 6% of body mass (BM) within 53 h. Energy deficit during the RWL was calculated by subtracting total energy expenditure (TEE) determined using the doubly labeled water method (DLW) from energy intake (EI) assessed with diet records. It was also estimated from body composition change estimated with the four-component model (4C) and other conventional methods. BM decreased significantly by 4.7 ± 0.5 kg (6.4 ± 0.5%). Total body water loss was the major component of the BM loss (71.0 ± 7.6%). TEE was 9446 ± 1422 kcal, and EI was 2366 ± 1184 kcal during the RWL of 53-h; therefore, the energy deficit was 7080 ± 1525 kcal. Thus, energy density was 1507 ± 279 kcal/kg ∆BM during the RWL, comparable with values obtained using the 4C, three-component model, dual energy X-ray absorptiometry, and stable isotope dilution. Energy density for RWL of wrestlers is lower than that commonly used (7400 or 7700 kcal/kg ΔBM). Although RWL is not recommended, we propose that commonly practiced extreme energy restriction such as 7400 or 7700 kcal/kg ΔBM during RWL appears to be meaningless.

## 1. Introduction

Optimal pre-competition weight management is essential for weight-classified sports such as wrestling, judo, and boxing. Weight-classified athletes are required to adjust their weight according to their weight-category until weigh-in, and occasionally undertake intense weight loss if needed. Methods of weight loss before and weight regain after weigh-in vary, and may be related to weigh-in methods, the amount of body mass (BM) loss, exercise and eating habits, the cultural context, season, level of competition, or sex [1,2]. The optimal duration of weight loss before competition is one of the major concerns of athletes, coaches, trainers, strength and conditioning specialists, and nutritional experts. The weight loss for weigh-ins for athletic competitions is sometimes associated with dehydration, resulting in dizziness, vertigo, and lightheadedness; this limits the ability to practice before the competition [3]. Several weight-classified athletes lose weight rapidly (>5%) during the final few days (2–3 days) before a competition after a long or intermediate period of mild weight loss [4].

Previous studies have reported changes in body composition and biomarkers after rapid weight loss (RWL) over 5–7 days [5,6,7,8,9]. For example, a study involving taekwondo athletes reported BM loss with 74% of change in fat-free mass (ΔFFM/ΔBM = 0.74) after 4 day of RWL assessed by bioelectrical impedance analysis (BIA) [9]. A further study measuring skinfold thickness in weightlifters reported BM loss (ΔFFM/ΔBM = 0.48) after 6 day of RWL [10]. However, most previous studies used BIA or skinfold methods, and thus there may have been errors in the estimation of body composition change. There is limited research on criterion techniques such as the four-component (4C) or three-component (3C) models. Currently, the 4C model is regarded as a criterion method for estimating body composition. However, no previous studies have examined the change of body composition during RWL within 1 week in weight-classified athletes using the 4C model. Moreover, it is unclear regarding the change of body composition and the required energy deficit for RWL over shorter durations (2–3 days) commonly employed by many combat athletes [4].

Energy loss during BM reduction is calculated as a difference between energy intake (EI) and total energy expenditure (TEE) during BM reduction. The doubly labeled water method (DLW) is regarded as the most accurate method to measure TEE in free-living subjects; however, DLW is costly, and the analysis is time-consuming. Therefore, a more cost-effective and convenient method is desirable for use in the sports field. The energy cost for BM reduction is assumed to be 7400 or 7700 kcal/kg ΔBM; however, some recent studies have indicated that these assumptions were not always suitable for BM change in athletes [11,12]. Body energy density is regarded as 1020 kcal/kg ∆FFM and 9500 kcal/kg ∆FM [13,14]. In theory, the energy cost for BM reduction varies depending on the ratio of ∆FFM/∆BM. Therefore, it is pivotal to measure changes in body composition during BM reduction accurately to obtain energy loss during BM reduction [15,16]. It is still unclear whether the 4C model and DLW can be applied to athletes undergoing RWL. Furthermore, it is important to evaluate whether other conventional methods for body composition measurement are accurate in the context of RWL. Since EI is calculated by subtracting energy loss from TEE, measuring both energy loss and TEE accurately should be helpful to organize EI during BM reduction.

The aims of the present study were (1) to measure the required energy deficit during RWL using the DLW method and EI (EI-TEE); (2) to assess whether energy balance was accurately estimated from body composition changes using the 4C model; and (3) to examine the accuracy of the energy balance estimated by body composition change using the 3C model, dual energy X-ray absorptiometry (DXA), stable isotope dilution (SID), air displacement plethysmography (ADP), and BIA. We hypothesized that the 4C model with current body energy density model could accurately assess energy balance during RWL. We postulated that the secondary indirect methods (e.g., BIA) would produce results with large errors in estimating body composition changes because these methods are affected by assumptions such as TBW distribution.

## 2. Materials and Methods

### 2.1. Subjects

Ten male collegiate wrestlers (age 20.4 ± 0.5 years, height 168.4 ± 4.2 cm, BM 73.0 ± 7.9 kg) were recruited from among universities in the Kanto area, Japan. The data of two subjects were excluded because they did not fulfill the criteria of merit (≤0.50 Hz) and airway pressure (<35.0 cmH_2_O) for thoracic gas volume (V_TG_) measurement during ADP measurement. Thus, a total of eight subjects were used for analysis. The following inclusion criteria were applied: experience of RWL involving over 6% of BM and competing at the international or national level or in the university wrestling championships in Japan. This study was approved by the Institutional Review Board of the Japan Institute of Sports Sciences (036). All subjects signed the appropriate informed consent forms before being evaluated.

### 2.2. Experimental Design

**Figure 1:**
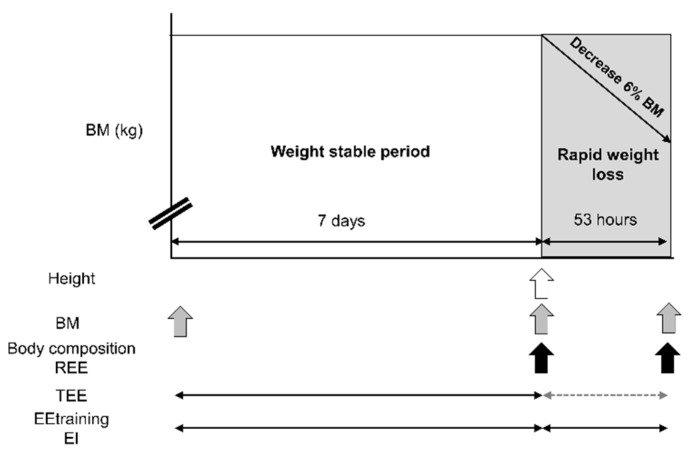
Experimental design. Abbreviations: BM, body mass; REE, resting energy expenditure; TEE, TEE during rapid weight loss indicated by dashed arrow was obtained by conversion of the TEE during the weight stable period into 53 h; EEtraining, energy expenditure during training calculated by rate of the perceived exertion with METs compendium; EI, energy intake.

The overall experimental design is described in Figure 1. Daily energy expenditure and daily energy and macronutrient intake during the preceding 7 days were measured in all subjects to determine their baseline levels. It has been reported that most of the athletes practicing RWL lose weight within 3 days [4]. We therefore instructed the subjects to lose 6% of their BM within 3 days, and they achieved the goal within 53 h after the PRE measurements. The weight loss methods were selected by each subject; for example: reducing food and fluid intake, sweating by using saunas, or training with plastic or rubberized suits. The method could be home- or club-based. The subjects were instructed to record all food and fluid intake and training procedures during the experimental period.

### 2.3. Anthropometry and Body Composition Measurements

The subjects refrained from consuming alcohol or stimulant beverages for at least 24 h prior to the baseline measurements, and they were instructed to abstain from consuming all food and beverages, except for water, after 23:00 of the day preceding the evaluation. The subjects presented to the laboratory at 06:30 and voided urine. Their heights were then measured to the nearest 0.1 cm with a stadiometer (A&D Co. Ltd., Tokyo, Japan). The subjects were weighed to the nearest 0.01 kg, using an electronic scale connected to the plethysmograph computer (BOD POD, COSMED, Rome, Italy); subjects were wearing a bathing suit and swim cap and were barefoot at the time of measurement. The PRE and POST values of body composition using ADP, BIA, DXA, SID, 4C model, and 3C model were determined during a single test session lasting approximately 6.5 h.

### 2.4. Air Displacement Plethysmography

ADP was utilized to measure body volume (BV) using BOD POD software (version 4.24. COSMED, Rome, Italy) in accordance with previous studies and previously described software [12,17]. Each subject wore a swimsuit and a swim cap, and the BV was measured on two or three occasions; V_TG_ measurement was then performed using a tube connected to the breathing circuit system. Body fat percentage (%fat) was calculated with the following equation [18].

%fat = (4.57/Db − 4.142) × 100(1)

### 2.5. Bioelectrical Impedance Analysis

BIA measurements were performed using the MC-980A analyzer (Tanita, Tokyo, Japan). The subjects stepped onto a platform where BM measurements were taken. Each subject then stood erect, took hold of the handles of the equipment, and pulled the arm down. The “Athlete Mode” was selected for the analysis of all wrestlers, as suggested by the manufacturer guidelines.

### 2.6. Dual-Energy X-ray Absorptiometry

Bone mineral content (Mo) and body composition were determined with whole-body scans by DXA (QDR 4500, Discovery A (S/N 84498), fan-beam scanner, software version 12.7.3.2, Hologic, Waltham, MA, USA). Wearing a swimsuit free of metal and/or plastic, each subject kept their body in the supine position for 5–10 min, according to the DXA protocol described by the manufacturer. The same technician positioned the participants, performed the scans, and analyzed body composition, according to the manual.

### 2.7. Stable Isotope Dilution

^2^H_2_O and H_2_^18^O were used to measure TBW at PRE and POST and TEE during the weight stable period. Urine and blood samples were obtained at 09:00. Participants drank ~0.12 g of ^2^H_2_O (^2^H_2_O 99.9 atom %; Taiyo Nippon Sanso, Tokyo, Japan) and ~1.5 g of H_2_^18^O (H_2_^18^O 20.0 atom %; Taiyo Nippon Sanso, Tokyo, Japan) per kg of their predicted TBW at PRE and ~0.06 g of ^2^H_2_O and ~0.75 g of H_2_^18^O per kilogram of their predicted TBW at POST. The TBW was predicted as 60% of initial BM. The bottle was rinsed twice with 30 mL of water. The participants drank 280 mL of bottled water within 3 h of dosing and emptied their bladders 2 h after dosing. Urine and blood samples were collected at 3 and 4 h after dosing. On day 4 (morning) and on day 7 (morning and evening) after PRE measurement, urine samples were collected, but only the samples obtained at 3 h and 4 h after dosing, and obtained in the morning and evening on day 7 were used for analysis to measure the TEE during the baseline period. The calculated 1-day TEE was converted to total 53-h energy expenditure as a TEE during RWL.

Aliquots of urine were frozen at −30 °C for later isotope ratio mass spectrometry (IRMS) analysis (Hydra 20-20 Stable Isotope Mass Spectrometers; Sercon Ltd., Crewe, UK). The equilibration gas for ^18^O was CO_2_ and that for ^2^H was H_2_. IRMS analysis was performed as previously described [19].

TBW volume was calculated from the plasma concentration of ^2^H and ^18^O according to the plateau method [20], adjusting for coefficient of ^2^H (1.041) and ^18^O (1.007) space because these isotopes enter other pools within the body and exchange with non-aqueous components [19,21]. FFM was calculated from TBW assuming a hydration of 0.73 [22].

TEE was calculated using Weir’s equation [23] based on rate of carbon dioxide production (rCO_2_) and the food quotient (FQ). The rCO_2_ in the baseline period was calculated using the two-point method [24]. The FQ estimated from the daily food record over 3 d (two training days and one non-training day) during the baseline period was 0.89 ± 0.02.

#### Three- and Four-Component Model

The 3C model used BM, BV, and TBW to calculate FM using the Siri equation [25], as described below:

FM = (2.118BV) − (0.78TBW) − (1.351BM)(2)

The 4C model used BM, BV, TBW, and Mo to calculate FM as described for adult subjects in previous reports [26]:

FM = (2.513BV) − (0.739TBW) + (0.947Mo) − (1.79BM)(3)

The densities of fat, water, bone mineral, and residual mass were assumed to be 0.900, 0.994, 2.982, and 1.34 g/cm^3^, respectively, for humans at 37 °C [27]. FFM was calculated as the difference between BM and FM.

### 2.8. Energy and Macronutrient Intake

A survey of all food and fluid intake was conducted before the baseline measurement and during the 53-h RWL period. The participants were provided with scales and instructed to weigh all consumed food, supplements, and beverages, and to take a photo with a ruler using a digital camera. The energy and macronutrient intake determined over 3 day included two training days and one day-off before the baseline measurement. A well-trained registered dietician calculated the EI and macronutrient intake from dietary records and photographs [28]. All dietary records were analyzed using a computerized nutrient analysis program (Excel Eiyou-kun Version 6.0, Japan food composition table Version 5, Kenpakusha, Tokyo, Japan). Energy and macronutrient content of foods that were commercially packaged or prepared at restaurants were calculated using the values on the manufacturer’s website or by asking the manufacturer.

### 2.9. Body Energy Density of Rapid Weight Loss

Body energy density of RWL was calculated from EI and TEE during the RWL period, and ∆BM [29] as follows:

Body energy density of RWL = (EI − TEE) kcal/kg ∆BM(4)

We also estimated the body energy density of RWL from a body composition change measured with each method [13,14]:

Body energy density of RWL = 1020 kcal/kg ∆FFM + 9500 kcal/kg ∆FM(5)

### 2.10. Resting Energy Expenditure and Energy Expenditure of Exercise and Training

The resting energy expenditure (REE) was measured using indirect calorimetry (ARCO-2000 MET and SYSTEM-5L; Arcosystem, Chiba, Japan), as described elsewhere [28]. The participants recorded details of the training program and the duration and rate of the perceived exertion during the entire measurement period. Energy expenditure during training (EE_training_) was estimated from the MET values [30] and duration of exercise practiced.

### 2.11. Statistical Analyses

All analyses were performed using SPSS version 24.0 (IBM, Tokyo, Japan), and are expressed as mean ± standard deviation. BM, body composition change using the 4C model, and energy and macronutrient intake were analyzed by paired *t*-test (PRE vs. POST). Analysis of variance (ANOVA) was used for repeated measures over time (PRE and POST) and for methodologies (4C model, 3C model, DXA, SID, ADP and BIA). The Bonferroni test was used for post-hoc analysis for between-methodology differences. Changes in %FM, FM and FFM, and the ratio of FM and FFM changes to BM changes were analyzed using one-way ANOVA with the Bonferroni post-hoc test. Energy density of RWL estimated with body composition changes after RWL obtained from DLW measurements and food records was compared using the Dunnett’s post-hoc test. By setting the statistical power (1−β error probability) at 0.8, the α error probability at 0.05, and the significant minimum effect size (Cohen’s d) at 1.2 (based on change of body composition [8,31]), our power calculation revealed that a minimum sample size of eight participants (for a paired *t*-test) was required to detect a statistically significant difference in the change in body composition.

## 3. Results

### 3.1. Body Mass and Body Composition

BM did not change during the weight-stable period (baseline 73.6 ± 7.7 kg, PRE 73.7 ± 8.0 kg), and significantly decreased by 4.7 ± 0.5 kg (6.4 ± 0.5%, *p* < 0.001) after RWL. TBW (*p* < 0.001) and fat-free dry solid weight (*p* < 0.01) significantly decreased after RWL (Table 1). However, there were no changes in Mo between PRE and POST.

**Figure 2:**
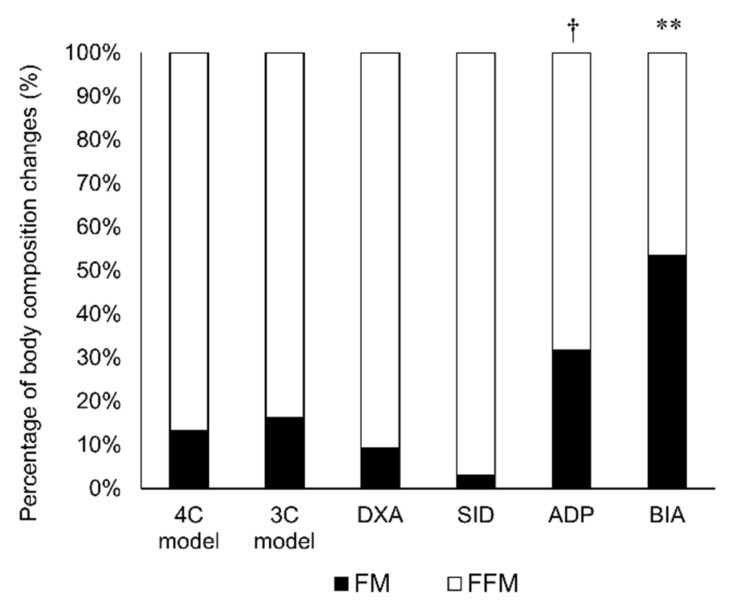
Ratio of body composition changes after rapid weight loss. Data are expressed as mean ± SD, *n* = 8. ** *p* < 0.01 vs. 4C model, 3C model, DXA, SID, and ADP using Bonferroni post-hoc test. † *p* < 0.05 vs. 4C model, 3C model, DXA, and SID using the Bonferroni post-hoc test.

As a result, FFM density significantly increased after RWL (*p* < 0.001). Statistical interaction (time × methodology) indicated different trajectories of %fat (*p* < 0.001), FM (*p* < 0.001) and FFM (*p* < 0.001), according to the methodologies (Table 2). No significant changes were observed in the %fat estimated by the 4C model, 3C model, and DXA between PRE- and POST-RWL measurements, whereas the %fat estimated by ADP (*p* < 0.01) and BIA (*p* < 0.001) significantly decreased. The %fat estimated from SID significantly increased (*p* < 0.05) after RWL. The changes in %fat and FM estimated by ADP and BIA were significantly greater than those estimated by the 4C model, 3C model, DXA, and SID method. The ratio of fat loss to BM loss estimated by ADP and BIA was significantly greater than that estimated by the 4C model, 3C model, DXA, and SID method (Figure 2).

### 3.2. Energy Balance During Rapid Weight Loss

**Figure 3:**
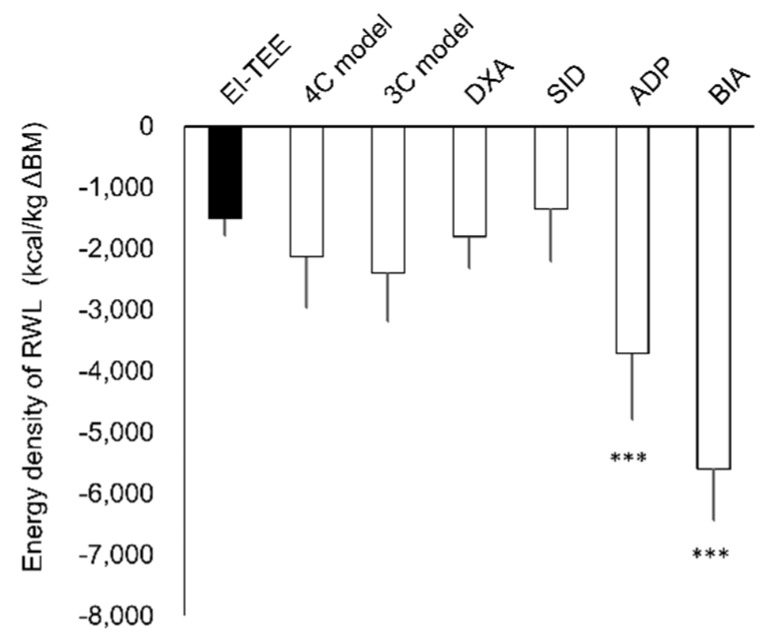
Energy density of rapid weight loss. Data are expressed as mean ± SD, *n* = 8. *** *p* < 0.001 vs. EI-TEE using Dunnett’s post-hoc test.

TEE was 4278 ± 644 kcal/day during baseline. REE (1696 ± 242 kcal/day) and EE_training_ (710 ± 202 kcal/day) during baseline remained unchanged during RWL (REE 1619 ± 164 kcal/day; EE_training_ 814 ± 168 kcal/day). TEE was 9446 ± 1422 kcal and EI was 2366 ± 1184 kcal during the RWL period; therefore, the energy deficit was 7080 ± 1525 kcal over the 53-h RWL period. Food weight/energy and macronutrient intake during the RWL period was significantly lower than at the baseline (Table 3). Energy density of RWL was 1507 ± 279 kcal/kg ∆BM (Figure 3). Furthermore, energy density of RWL calculated with EI-TEE did not differ from the values calculated using the 4C model, 3C model, DXA, and SID methods. However, energy density of RWL estimated using the ADP and BIA methods was significantly greater than that of EI-TEE.

## 4. Discussion

The novel findings of the present study are an accurate determination of the energy deficit during 53-h RWL using the EI-TEE measurements, using the DLW methods and the 4C model to represent the criterion for measuring energy balance and body composition, respectively. In addition, no differences were observed in the estimated changes in %fat, FM, and FFM for the 3C model, the DXA, and the SID method compared with the measured EI-TEE. In contrast, the ADP- and BIA-estimated FM loss was greater than that estimated by the 4C model. A body energy density model with 1020 kcal/kg ∆FFM and 9500 kcal/kg ∆FM indicated by previous literature can be applied to the RWL situation. During RWL for the athletes, the loss of BM mostly depended on loss of TBW (71.0 ± 7.6%), and the energy density of RWL was only 1507 kcal/kg ∆BM.

We aimed to determine a method for the accurate measurement of energy balance during RWL. We thus examined accuracies of the 4C model, which is a criterion method of estimating human body composition, and the DLW method, which is a criterion method of measuring TEE in free-living settings. In recent years, DLW and the 4C model have been used in energy balance and body composition change studies in athletes [12,32]. Silva et al. [12] examined 16-week body composition changes in athletes and reported that the ratios of FFM and FM changes to BM changes were 15% and 85%, respectively, with weight loss >1.5% in baseline BM, ranging from −7.0 to −1.5% BM. They also stated that the energy density of weight loss was 8182 kcal/kg ∆BM. Bhutani et al. [11] reported that the average energy density of more short-term (2-week) fluctuations in BM of 1–3 kg was 2380 kcal/kg ∆BM in healthy adults. Our energy density of RWL (1507 kcal/kg) were close to the latter study. Therefore, the period and magnitude of weight loss is a possible factor affecting the ratio of body composition change and the energy density of ∆BM during weight loss. Previous studies reported that the percentage of BM reduction by FFM loss (ΔFFM/ΔBM) was higher during RWL compared with slow weight loss [33,34]. Yang et al. [9] compared 4-day and 4-week weight loss in taekwondo athletes. Both groups decreased 5% of BM, and the 4-day group had no significant change in FM, while the 4-week group significantly decreased their FM. The ΔFFM/ΔBM was 0.74 in the 4-day rapid BM loss group and 0.27 in the 4-week, moderate-duration weight loss group. In the present study, the ΔFFM/ΔBM was 0.87, which was much greater than the value (ΔFFM/ΔBM = 0.53) calculated from Forbes’s equation [33]. RWL associated with dehydration induces a decrease in glycogen [35,36], and water loss in fat-free tissue causes dehydration [8,37]; therefore, the percentage of FFM reduction against BM reduction during RWL is greater than that in moderate-speed weight loss programs. Therefore, the energy density of RWL for 1 kg BM was much smaller compared with normal weight loss programs.

Sagayama et al. [8] reported that the ratio of ΔFFM/ΔBM after 5% RWL in 1 week was 0.66 among athletes whose baseline %fat was 17.0 ± 7.4% determined with the 3C model. We calculated ΔFFM/ΔBM after BM loss from body composition changes in a range of athletes in the literature as follows: 0.52 in judo athletes (baseline %fat, 13.4%) after a 5-day weight loss program [5], 0.42 in weightlifters (baseline %fat, 22.2%) after a 6-day weight loss program [10], and 0.70 among combat athletes (baseline %fat, 12.5%) after 5–7 day of weight loss [6]. These previous studies measured %fat using various methods (e.g., skinfold method), so caution should be exercised in directly comparing these studies, but the subjects in the present study had lower baseline %fat (12.4 ± 2.5%) than those in previous studies [5,6,8,10]. According to Forbes’s equation, participants with lower %fat at baseline tend to lose weight with a greater ratio of ΔFFM/ΔBM [29,33,34]. The low baseline %fat (12.4%) in the present subjects might be associated with a greater decrease in the ratio of ΔFFM/ΔBM compared with the previous studies. However, our result of ΔFFM/ΔBM was much greater than the value estimated from the Forbes’s equation [33]. These results suggest that the ratio of ΔFFM/ΔBM during RWL in weight-classified athletes depends on their weight loss duration as well as their baseline %fat.

This study has some limitations. The DLW method can be used to evaluate TEE accurately over 1–2 weeks; however, this method cannot be used for shorter periods (<3 day). Therefore, we measured 7-day TEE before weight loss periods, and then applied the value for the RWL period. Previous studies indicate that REE [38] or sleeping metabolic rate [8] decrease after weight loss programs lasting >1 week. In the present study, however, there were no differences between baseline and the RWL period in REE and EE_training_. Furthermore, the duration of RWL in the current study was very short (53 h). Müller et al. [38] observed changes in REE, adaptive thermogenesis and respiratory quotient from the third day of calorie restriction. Therefore, the difference in TEE seems to be small between the baseline 7-day period and the 53-h RWL period. Another limitation is that the EI might be underestimated. We instructed all participants to weigh, record and photograph all foods and drinks and to record or photograph the nutrition and ingredient labels of commercially prepared foods that they consumed. In addition, the participants consumed fewer food items than usual to reduce energy intake, which made it easier to calculate energy intake. Therefore, it is considered that despite limitations the energy balance was estimated accurately.

## 5. Conclusions

In conclusion, we observed that the energy density of 53-h RWL was 1507 kcal/kg ΔBM. This was because the BM loss was mostly achieved by loss of TBW. Although RWL is not recommended, we propose that commonly practiced extreme energy restriction such as 7400 or 7700 kcal/ΔBM during RWL appears to be meaningless. Our findings show that the energy deficit calculated with the 4C model based on 9500 kcal/kg ΔFM and 1020 kcal/kg ΔFFM is consistent with that evaluated using TEE measured with DLW and EI under this RWL situation. Furthermore, the energy density of weight loss could be estimated accurately using the 3C model, DXA, and SID, whereas ADP and BIA are not advisable methods for assessing body composition during RWL.

## Figures and Tables

**Table 1:** Participant characteristics and change of body composition using the 4C model with stable isotope dilution method and DXA.

	PRE	POST	Change
Age (years)	20.3 ± 0.5	-	-
Height (cm)	169.7 ± 3.5	-	-
BM (kg)	73.7 ± 8.0	69.0 ± 7.7 ***	−4.7 ± 0.5
%fat	12.4 ± 2.5	12.3 ± 2.5	−0.1 ± 0.6
FM (kg)	9.1 ± 1.9	8.5 ± 1.8 **	−0.6 ± 0.4
FFM (kg)	64.6 ± 7.4	60.5 ± 6.8 ***	−4.1 ± 0.8
TBW (kg)	46.8 ± 5.6	43.5 ± 5.2 ***	−3.4 ± 0.6
TBW (%/kg FFM)	72.5 ± 0.7	71.9 ± 0.6 *	−0.6 ± 0.4
Mo (kg)	3.2 ± 0.3	3.2 ± 0.3 *	0.0 ± 0.0
Mo (%/kg FFM)	5.0 ± 0.3	5.3 ± 0.4 ***	0.4 ± 0.0
FFDS (kg)	14.5 ± 1.6	13.8 ± 1.4 **	−0.8 ± 0.4
FFDS (%/kg FFM)	22.5 ± 0.5	22.8 ± 0.3	0.2 ± 0.4
Density of FFM (g/cm^3^)	1.094 ± 0.003	1.098 ± 0.004 ***	0.004 ± 0.001

Data are expressed as means ± SD, *n* = 8. * *p* < 0.05, ** *p* < 0.01, *** *p* < 0.001 vs. PRE using paired *t*-test. Abbreviations: BM, body mass; %fat, percentage of fat mass; FM, fat mass; FFM, fat free mass; TBW, total body water; Mo, bone mineral content; FFDS, fat free dry solid.

**Table 2:** Differences in body composition changes per method used.

	Methods	PRE	POST	Change
%fat	4C model	12.4 ± 2.5	12.3 ± 2.5	−0.1 ± 0.6 ^a^
	3C model	12.8 ± 2.4	12.5 ± 2.2	−0.2 ± 0.5 ^a^
	DXA	11.1 ± 1.8	11.2 ± 2.1	0.1 ± 0.4 ^a^
	SID	13.2 ± 2.7	13.9 ± 2.4 **	0.7 ± 0.6 ^a^
	ADP	11.7 ± 2.4	10.3 ± 2.3 ***	−1.4 ± 0.9 ^b^
	BIA	13.3 ± 2.0	10.4 ± 2.5 ***	−2.8 ± 0.9 ^c^
Fat mass (kg)	4C model	9.1 ± 1.9	8.5 ± 1.8 **	−0.6 ± 0.4 ^a^
	3C model	9.4 ± 1.9	8.6 ± 1.8 ***	−0.7 ± 0.4 ^a^
	DXA	8.2 ± 1.5	7.7 ± 1.6 **	−0.4 ± 0.3 ^a^
	SID	9.7 ± 1.9	9.6 ± 1.7	−0.1 ± 0.4 ^a^
	ADP	8.7 ± 2.2	7.2 ± 2.1 ***	−1.5 ± 0.6 ^b^
	BIA	9.8 ± 2.2	7.2 ± 2.3 ***	−2.5 ± 0.6 ^c^
Fat free mass (kg)	4C model	64.6 ± 7.4	60.5 ± 6.8 ***	−4.1 ± 0.8 ^ab^
	3C model	64.3 ± 7.3	60.3 ± 6.7 ***	−4.0 ± 0.7 ^ab^
	DXA	65.5 ± 7.2	61.3 ± 7.0 ***	−4.3 ± 0.4 ^ab^
	SID	64.0 ± 7.6	59.4 ± 7.1 ***	−4.6 ± 0.8 ^a^
	ADP	65.0 ± 7.0	61.8 ± 6.3 ***	−3.2 ± 0.8 ^bc^
	BIA	63.5 ± 6.4	61.3 ± 6.2 ***	−2.2 ± 0.4 ^c^

Data are expressed as mean ± SD, *n* = 8. Significant interaction between time and methods are indicated by repeated measures ANOVA. ** *p* < 0.01, *** *p* < 0.001 showed main effects (time). Values of the change marked by different superscript letters within the column were different significantly different according to the Bonferroni post-hoc test, *p* < 0.05.

**Table 3:** Food weight, energy and macronutrient intake and energy expenditure.

		Baseline	RWL Period
Food weight	(g/day)	3686 ± 1615	1464 ± 720 **
Energy intake	(kcal/day)	3528 ± 829	1071 ± 536 ***
	(kcal/kg BM/day)	47.9 ± 10.2	14.5 ± 6.8 ***
Protein	(g/day)	125 ± 30	38 ± 18 ***
	(g/kg BM/day)	1.7 ± 0.4	0.5 ± 0.2 ***
	(%energy)	14.2 ± 0.9	14.6 ± 2.8
Fat	(g/day)	110 ± 24	34 ± 20 ***
	(g/kg BM/day)	1.5 ± 0.3	0.5 ± 0.3 ***
	(%energy)	28.7 ± 5.3	28.4 ± 9.3
Carbohydrate	(g/day)	509 ± 149	154 ± 77 ***
	(g/kg BM/day)	6.9 ± 1.9	2.1 ± 1.0 ***
	(%energy)	57.1 ± 5.4	57.0 ± 11.4

Data are expressed as means ± SD, *n* = 8. ** *p* < 0.01, *** *p* < 0.001 for the difference from baseline using paired *t*-test.
